# "The West London Appointment": an Official Disclaimer

**Published:** 1910-12-31

**Authors:** Arthur Saunders

**Affiliations:** Chairman of the Medical Staff and Physician to the Hospital.


					Dear Sir,?The medical staff of the West London
Hospital wish to dissociate themselves from the paragraph
appearing in your issue of December 17 under the heading
" A West London Appointment."
This paragraph implies that the selection of the Medical
Council was overridden by the Board of Management.
We beg to state that the candidate appointed was one of
three selected by the Medical Council and recommended
for election. So far from there being any dissatisfaction
on the part of the medical staff, they have warmly wel-
comed their new colleague.?Yours faithfully,
Arthur Saunders,
Chairman of the Medical Staff and
Physician to the Hospital."
[We refer to this letter in "Week's Work."?Editor,
The Hospital.]

				

